# Editorial: Chloroplast Biotechnology for Crop Improvement

**DOI:** 10.3389/fpls.2022.848034

**Published:** 2022-02-01

**Authors:** Clelia De-la-Peña, Patricia León, Thomas D. Sharkey

**Affiliations:** ^1^Unidad de Biotecnología, Centro de Investigación Científica de Yucatán, Mérida, Mexico; ^2^Instituto de Biotecnología Universidad Nacional Autónoma de Mexico, Cuernavaca, Mexico; ^3^MSU-DOE Plant Research Laboratory, Michigan State University, East Lansing, MI, United States; ^4^Department of Biochemistry and Molecular Biology, Michigan State University, East Lansing, MI, United States; ^5^Plant Resilience Institute, Michigan State University, East Lansing, MI, United States

**Keywords:** chloroplast, crops, biotechnology, editorial, photosynthesis

Ever since the first photosynthetic eukaryote organisms appeared, chloroplasts have been an enigmatic cellular compartment where complex biochemical and molecular processes essential for the plant take place. As a result, substantial efforts in plant biotechnology have been linked to improve photosynthetic efficiency and also the production of a diversity of important compounds synthesized in this organelle that are of biological, medical and industrial importance.

Chloroplast biotechnology may open new frontiers of crop development (Maliga and Bock, 2011) having broad impacts on medicine, fuel, food, bioplastics, and chemicals and also may help mitigate deleterious effects of global warming on plant function and productivity. Therefore, it is of central importance to study and investigate the molecular mechanisms that chloroplasts use and that can be exploited by crop biotechnology in the future. Understanding how chloroplasts work will open opportunities for modifications and improvement that would benefit our modern society.

This Research Topic includes an excellent combination of Reviews and Original Research Articles focused on *Chloroplast Biotechnology for Crop Improvement*, and provides up-to-date information on chloroplast genome sequencing, photosynthesis improvement, chloroplast metabolic engineering, and plastid-related metabolite production, using new approaches and novel technologies.

One of the biotechnological tools for crop improvement has been the use of high-throughput technologies such as the newest-generation sequencing to obtain genome sequence information, the use of nanotechnology for plastid transformation, and genetic engineering. For instance, in their Original Research article Yu et al. reported the complete chloroplast genome of an important medicinal plant *Erigeron breviscapus* identifying regulatory elements and providing a foundation for the establishment of the subsequent chloroplast genetic transformation system for genetic improvement. On the other hand, specific topics on the role of nanotechnology are discussed by Newkirk et al. providing interesting new insights and up to date status into this field. In their Review, they highlight recent discoveries on the interactions between chloroplasts and nanomaterials, nanomaterial uses for crop performance monitoring and improvement and nanotechnological applications for plastid synthetic biology that enable chloroplast uses for manufacturing. These tools may allow increased plant productivity and make sustainable technologies widely available.

It is known the potential risk of contaminating genetically modified (GM)-free cropland with GM pollen or seed can be increased if GM plants are cultivated in areas where there are wildtype relatives of the respective GM plant present. Alternatively, the integration into the plastid genome of the genes of interest can avoid the risk of GM pollen spread and sterility technologies may prevent the genetic exchange altogether (Clark and Maselko, 2020). Since chloroplast transformation has many advantages over the classic genomic transformation such as high expression and transgene safety, identification of high efficiency regulatory elements is central for developing and improving efficient chloroplast transformation in diverse species. In their Research Topic Shahinnia et al. offer a convincing case for engineering the photosynthetic electron transport chain with heterologous proteins, aimed to increase barley productivity. Furthermore, Moreno et al. found that the manipulation of carotenoid metabolism results in higher biomass production under normal cultivar conditions, with improved stress tolerance bearing no negative impact on the overall plant growth. In their Research Topic Moreno et al. take advantage of a multi-OMICs (transcriptomics, proteomics, metabolomics, and lipidomics) approaches to deeply characterize the phenotype of transgenic tobacco plants overexpressing the carrot lycopene β-cyclase 1 (*LCYB1*) gene. This study will certainly help in the future identification of transcription factors associated with stress responses.

Photosynthetic carbon assimilation has two major problems: stomatal density and CO_2_ levels, and different authors address these challenges in crop productivity. Sakoda et al., using different *Arabidopsis* genotypes that differ in stomatal density, find that most of the effects of stomatal density are on photosynthesis, CO_2_ assimilation rate, and biomass production. The authors also demonstrated that a short-term effect on photosynthesis rate measured per unit leaf area can well translate to significant whole-plant growth effects. Also, the effect of CO_2_-concentrating mechanisms was highlighted in the Review of Rottet et al., who give an overview of the major candidate proteins to be considered for engineering carbon concentrating mechanisms into C_3_ chloroplasts and highlight the complexities associated with the heterologous expression and generation of functional ${\text{HCO}}_{3}^{-}$ transport systems in C_3_ chloroplasts for increased Rubisco carboxylation rate and thereby for higher photosynthetic rates.

Understanding the control mechanisms underlying carbon partitioning is critical for engineering starch content and metabolic flux in plants (Santelia and Zeeman, 2011; Kölling et al., 2015). In the Research Topic of Preiser et al., kinetic properties of plastidic and cytosolic isoforms of phosphoglucoisomerase in the presence and absence of different regulatory factors was characterized. This is an important question for exploring plant carbon partitioning dynamics, which vary with growth, environmental conditions, metabolic state, and the circadian clock.

High-value nutritional compounds and therapeutic proteins have been obtained from chloroplasts. Jackson et al. review current knowledge on *Chlamydomonas* chloroplast system as a potential tool for testing synthetic biology strategies, which could be further translated in higher plants. In their Review, Jackson et al. describe some of the radical synthetic biology (SynBio) ideas and Design-Build-Test-Learn (DBTL) strategies highlighting the speed and versatility of *Chlamydomonas* chloroplast genetic engineering for improving photosynthesis, carbon fixation, nitrogen fixation, and production of novel metabolites. Arias et al. investigate the effect of the direct expression of transgenes phytoene synthase (*DcPSY2*) and lycopene cyclase (*DcLCB1*) from *Daucus carota* and carotene desaturase (*XdCrtI*) from yeast *Xanthophyllomyces dendrorhous* in tomato and apple fruits. They find a clear increase in the β-carotene and carotenoid levels demonstrating the potential use of these genes for biotechnological applications. This kind of Research Topic will certainly help to increase the nutritional value in fruits.

On the other hand, in their Research Topic, Hung et al. show that a long-lived albino mutant can be used as a model for analyzing the function of chloroplast-development genes and facilitate the purification of therapeutic proteins without chlorophyll interference. Vaccine production is one of most intensively studied fields in molecular farming. The production of proteins in chloroplasts can considerably reduce the costs of producing proteins focused on the pharmaceutical area. Whereas, many scientists have developed different systems to produce large amounts of vaccines, Nakahira et al. reported in their Research Topic, chloroplast transformation to optimize a high production of recombinant protein in transgenic plants without the risk of gene transfer through pollen distribution, i.e., *via* maternal inheritance. They reported the production of virus-like particles (VLPs) of red-spotted grouper nervous necrosis virus (RGNNV) in tobacco plants using chloroplast transformation techniques.

The Research Topic presented here is significant because it is expected to increase and strengthen the information needed to develop, in the near future, novel approaches to manipulate and selectively activate chloroplast development to increase crop productivity. New approaches of the type presented here, and the advancement of new technologies will certainly increase our knowledge of currently known photosynthetic organisms and will facilitate the understanding of their roles as sources of sustainable foods, novel biopharmaceuticals, and next-generation biomaterials essential for modern society.

## Author Contributions

CD-l-P, PL, and TS provided the idea of the work and critically reviewed the manuscripts. CD-l-P wrote the paper. All authors read and approved the final manuscript and contributed to the article and approved the submitted version.

## Funding

This work was supported by CONACYT grants CB-2016 # 285898 and # 286368 to CD-l-P. DGAPA-UNAM IN207320 for PL. TS was supported by the Division of Chemical Sciences, Geosciences and Biosciences, Office of Basic Energy Sciences of the United States Department of Energy Grant DE-FG02-91ER20021 and receives partial salary support from MSU AgBioResearch.

## Conflict of Interest

The authors declare that the research was conducted in the absence of any commercial or financial relationships that could be construed as a potential conflict of interest.

## Publisher's Note

All claims expressed in this article are solely those of the authors and do not necessarily represent those of their affiliated organizations, or those of the publisher, the editors and the reviewers. Any product that may be evaluated in this article, or claim that may be made by its manufacturer, is not guaranteed or endorsed by the publisher.
